# Appearance of a transparent protrusion containing two pairs of legs on the apodous ring preceding the anamorphic molt in a millipede, *Niponia nodulosa*

**DOI:** 10.1186/s12983-023-00493-0

**Published:** 2023-04-18

**Authors:** Soma Chiyoda, Kohei Oguchi, Toru Miura

**Affiliations:** Misaki Marine Biological Station, School of Science, The University of Tokyo, Misaki, Miura, Kanagawa 238-0225 Japan

**Keywords:** Arthropod, Millipede, Segment, Appendage, Anamorphosis, Molt, Morphogenesis, Postembryonic development

## Abstract

**Background:**

Arthropods gradually change their forms through repeated molting events during postembryonic development. Anamorphosis, i.e., segment addition during postembryonic development, is seen in some arthropod lineages. In all millipede species (Myriapoda, Diplopoda), for example, postembryonic processes go through anamorphosis. Jean-Henri Fabre proposed 168 years ago the “law of anamorphosis”, that is, “new rings appear between the penultimate ring and the telson” and “all apodous rings in a given stadium become podous rings in the next stadium”, but the developmental process at the anamorphic molt remains largely unknown. In this study, therefore, by observing the morphological and histological changes at the time of molting, the detailed processes of leg- and ring-addition during anamorphosis were characterized in a millipede, *Niponia nodulosa* (Polydesmida, Cryptodesmidae).

**Results:**

In the preparatory period, a few days before molting, scanning electron microscopy, confocal laser scanning microscopy, and histological observations revealed that two pairs of wrinkled leg primordia were present under the cuticle of each apodous ring. In the rigidation period, just prior to molt, observations of external morphology showed that a transparent protrusion was observed on the median line of the ventral surface on each apodous ring. Confocal laser scanning microscopy and histological observations revealed that the transparent protrusion covered by an arthrodial membrane contained a leg bundle consisting of two pairs of legs. On the other hand, ring primordia were observed anterior to the telson just before molts.

**Conclusions:**

Preceding the anamorphic molt in which two pairs of legs are added on an apodous ring, a transparent protrusion containing the leg pairs (a leg bundle) appears on each apodous ring. The morphogenetic process of the rapid protrusion of leg bundles, that is enabled by thin and elastic cuticle, suggested that millipedes have acquired a resting period and unique morphogenesis to efficiently add new legs and rings.

## Background

So far, many evo-devo studies in arthropods have focused on the mechanisms of segmentation and appendage formation, providing us with a number of important questions about the body-plan evolution [1–3]. Arthropods gradually change their forms through repeated molting events during postembryonic development. In arthropods, two developmental modes are known, anamorphosis and epimorphosis [4–7]. In anamorphosis, new segments are added with molting, that is, segmentation and appendage formation are not completed during embryogenesis, but continue through postembryonic development (e.g., sea spiders, copepods, remipedes, proturans) [8–11]. On the other hand, in epimorphosis, as seen in insects, the numbers of segments and legs are determined at the time of hatching and remain constant throughout postembryonic development. This means that segmentation and appendage formation are completed during embryogenesis. Furthermore, anamorphosis is classified into three types based on the patterns of molting and segment addition [6]. In hemianamorphosis, the addition of new segments and legs goes on until a certain instar, and then further moltings take place without the addition of segments or legs. In other words, this mode involves first anamorphosis, and secondly, epimorphosis. In euanamorphosis, every molting is accompanied by the addition of new segments and legs, even beyond the acquisition of sexual maturity. There is no limit to the number of moltings, and the addition continues until the death of the animal. In teloanamorphosis, every molting is accompanied by the addition of new segments and legs, but further molting does not occur after reaching adulthood. Among the three, hemianamorphosis is considered the plesiomorphic mode in arthropods [12, 13]. Since epimorphosis is thought to have derived from anamorphosis [7], investigations on anamorphosis would be important for understanding the ancestral pattern of segmentation and appendage formation in arthropods. However, information about morphogenetic processes involved in anamorphosis is scarce.

In particular, the subphylum Myriapoda has been less studied than other arthropods, e.g., insects, and knowledge about its developmental processes is limited. Since modes of postembryonic development are remarkably diversified in myriapods, elucidation of these processes in myriapods would provide insights into the evolution of body-plan diversity in arthropods. Myriapoda consists of four classes: Chilopoda (centipedes), Diplopoda (millipedes), Pauropoda (pauropods), and Symphyla (symphylans) [14]. In Myriapoda, all four modes of postembryonic development (epimorphosis, hemianamorphosis, euanamorphosis, and teloanamorphosis) have been observed [6, 15]. Hemianamorphosis is regarded as the ancestral mode [12, 13]. It is considered that enanamorphisis and teloanamorphosis evolved from hemianamorphosis after diversification of the infraclass Helminthomorpha (class Diplopoda) and epimorphosis was derived from the hemianamorphic chilopod lineage [16].

The body of an arthropod consists of a "segment" that is a serially homologous body unit with a pair of appendages. The terminology of the segment is somewhat complicated in millipedes. In ring-forming millipedes, the body ring which is formed by the fusion of tergite, pleurite and sternite does not correspond to a “true” segment [17–19]. The first tergite with no leg pairs, i.e., collum, can be regarded as the first ring although it is not a complete ring, so that the following rings are often termed as the second, third, and fourth rings, each of which has a single pair of legs, therefore called “haplorings.” Each of the fifth and subsequent rings has two pairs of legs, because it is thought to have derived from the fusion of two segments, therefore called “diplorings” [18, 19]. In this paper, therefore, the term "ring" is used to discuss a body unit in millipedes.

A French entomologist Fabre observed the postembryonic development of *Polydesmus complanatus* (Polydesmida, Polydesmidae) and described it as “Chaque nouvel anneau apparaît entre l'avant-dernier et l'anneau anal.” (Each new ring appears between the penultimate and the telson) and “Tous les anneaux apodes d'un stade deviennent pédigères au stade suivant.” (All apodous rings in a given stadium, i.e., instar, become podous rings in the next stadium) [20]. This pattern has been called the “law of anamorphosis” and this law was shown to be applicable to some other millipede species (e.g., [21]). So far, this law is known to be applicable to the three millipede groups: (1) the ring-forming groups (Polydesmida and Juliformia), (2) Chordeumatida, and (3) at least some Polyzoniida [6]. However, the morphogenetic processes leading to the ring and leg addition at the time of molt are largely unknown. In the hothouse millipede, *Oxidus gracilis* (Polydesmida, Paradoxosomatidae), just before molting, a “leg bud” that will give rise to each newly added leg appears [22], although the developmental process has yet to be elucidated. Therefore, in this study, to clarify the processes of the ring and leg addition following the law of anamorphosis, observations of the morphological changes during molting were carried out in a millipede, *Niponia nodulosa* (Polydesmida, Cryptodesmidae)*.*

**Fig. 1:**
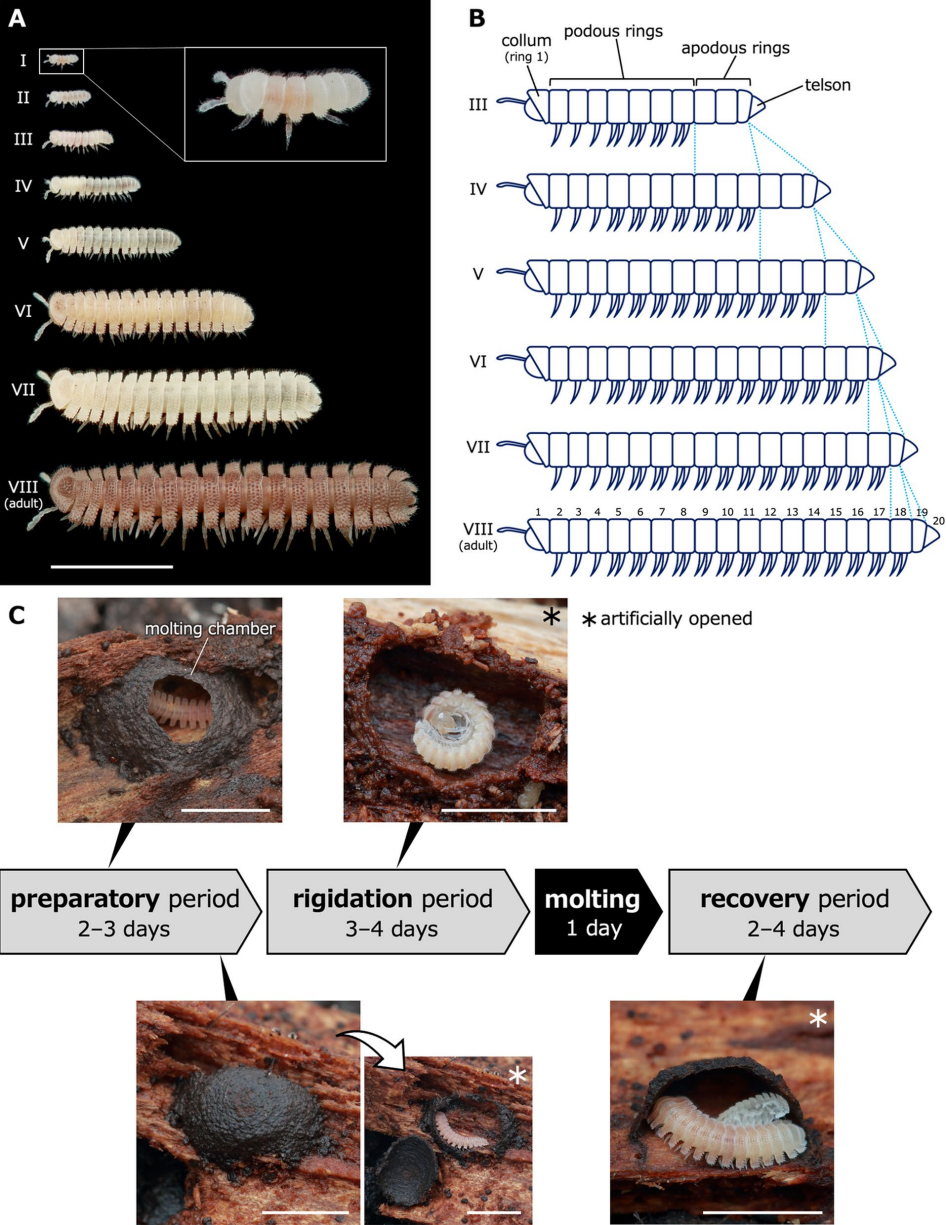
Patterns of postembryonic development and molting behaviors in *Niponia nodulosa*, with reference to Shinohara, 1999 [25]. **A** Life stages of *N. nodulosa*. **B** A diagram showing the pattern of postembryonic development of stadium III to VII juveniles and adults. **C** Behavioral series at the time of molting, showing the representative pattern of molting from stadium V to VI juveniles. The same pattern is also seen in other molts. Scale bars show 5 mm

The postembryonic developmental schedules have been described for some polydesmids (Polydesmida), namely, *Oxidus gracilis* [22], *Ampelodesmus iyonis* (Pyrgodesmidae) [23], and *Xystodesmus gracilipes* (Xystodesmidae) [24]. However, all of these previous studies only clarified the schedule and pattern of the anamorphosis, and no detailed morphological and histological observations were performed at the anamorphic molt. All polydesmids are known to develop by teloanamorphosis. *Niponia nodulosa* is known to develop by teloanamorphosis, like other polydesmid species. Stadium I, II, III, IV, V, VI, VII juveniles and stadium VIII (adults) have respectively 7, 9, 12, 15, 17, 18, 19, and 20 rings (Fig. 1A) [25]. Based on the description of the postembryonic development, in molts from at least stadium III, it appears that all apodous rings in a given stadium become podous rings in the next stadium and new rings are added between the penultimate ring and the telson (Fig. 1B). The law of anamorphosis can also be applied to *N. nodulosa*. The following behaviors are observed in *N. nodulosa* at the time of molt (Fig. 1C) [25]. Prior to each molt, juveniles of *N. nodulosa* construct molting chambers in which they molt to the subsequent stadium. The period from the start of the molting chamber construction until the molting chamber is completely constructed is defined as the preparatory period. Even at the late preparatory period when the molting chamber has been completely constructed, the millipede in it still can move. The rigidation period is defined as the period when the millipedes curl up and become motionless. The time required for each period is 2–3 days for the preparatory period and 3–4 days for the rigidation period. After the molting, i.e., exuviation, the millipedes remain in the molting chamber for 2–4 days until they are able to move sufficiently, which is defined as the recovery period.

In this study, detailed stereomicroscopy of live juveniles of the focal species at the late preparatory period and the rigidation period was performed, in addition to scanning electron microscopy, confocal laser scanning microscopy, and histological observations. Stadium IV–VII juveniles were used because the law of anamorphosis fit their developmental patterns well. For comparison, stereomicroscopy and scanning electron microscopy observation of *Epanerchodus* sp. (Polydesmida, Polydesmidae) were performed.

## Results

### External morphology at the time of molt

**Fig. 2:**
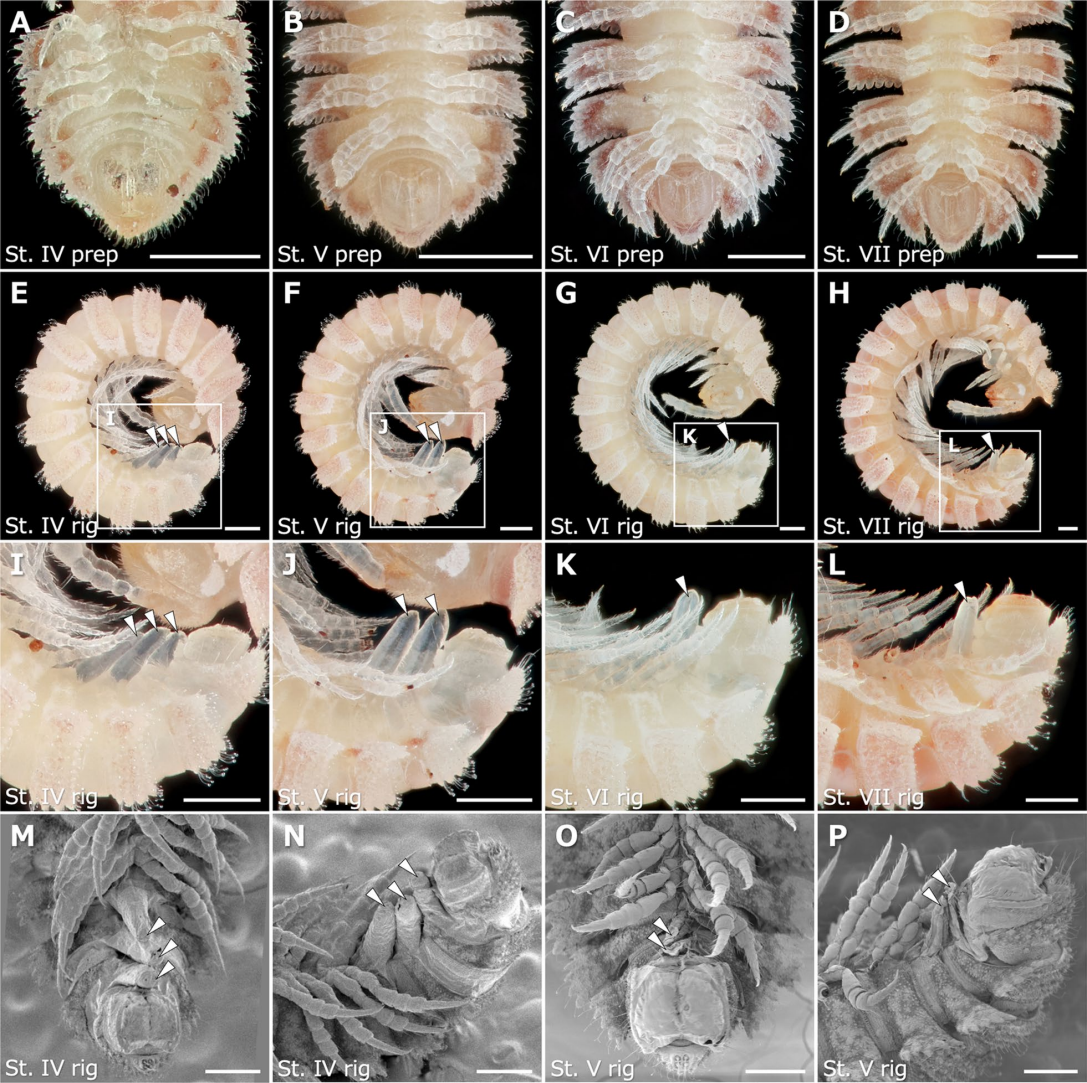
External morphologies of juveniles in the late preparatory period and the rigidation period. **A**–**D** Ventral view of the posterior several rings in the late preparatory period. **E**–**H** Lateral view in the rigidation period. **I**-**L** Higher magnifications of the white boxed regions in **E**–**H**. **M**–**P** Scanning electron microscopy images in the rigidation period. **M**,**O** Ventral view of the posterior several rings. **N**,**P** Lateral view of the posterior several rings. Ventral is up. **A**,**E**,**I**,**M**,**N** Stadium IV juveniles. **B**,**F**,**J**,**O**,**P** Stadium V juveniles. **C**,**G**,**K** Stadium VI juveniles. **D**,**H**,**L** Stadium VII juveniles. Arrowheads: a transparent protrusion. Scale bars show 500 μm (**A**–**L**) and 200 μm (**M**–**P**)

To elucidate morphological changes at the time of molt to the next stadium, observations were performed on external morphologies of juveniles at the late preparatory and the rigidation period. In stadium IV–VII juveniles at the late preparatory period, no distinct primordial structure was found on the ventral surface of the apodous rings (Fig. 2A–D). In stadium IV–VII juveniles at the rigidation period, transparent protrusions were observed on the ventral surface of the tail ends (Fig. 2E–L). The number of transparent protrusions corresponded to the number of apodous rings. Namely, 3, 2, 1 and 1 protrusions were observed respectively at the end of stadium IV, V, VI and VII juveniles.

Based on SEM observations on stadium IV and V juveniles at the rigidation period, each transparent protrusion was seen on rings between an apodous ring and the posterior ring on the median line (Fig. 2M–P). In stadium IV juveniles, for example, the most anterior protrusion was observed between the most anterior apodous ring and the posterior one, the second protrusion was observed between the second apodous ring and the posterior one, and the most posterior protrusion was observed between the most posterior apodous ring and the telson (Fig. 2M, N). Each protrusion was covered with a thin membrane, which was a part of the membrane connecting an apodous ring with the posterior one.

### Histological observations at the time of molt

**Fig. 3:**
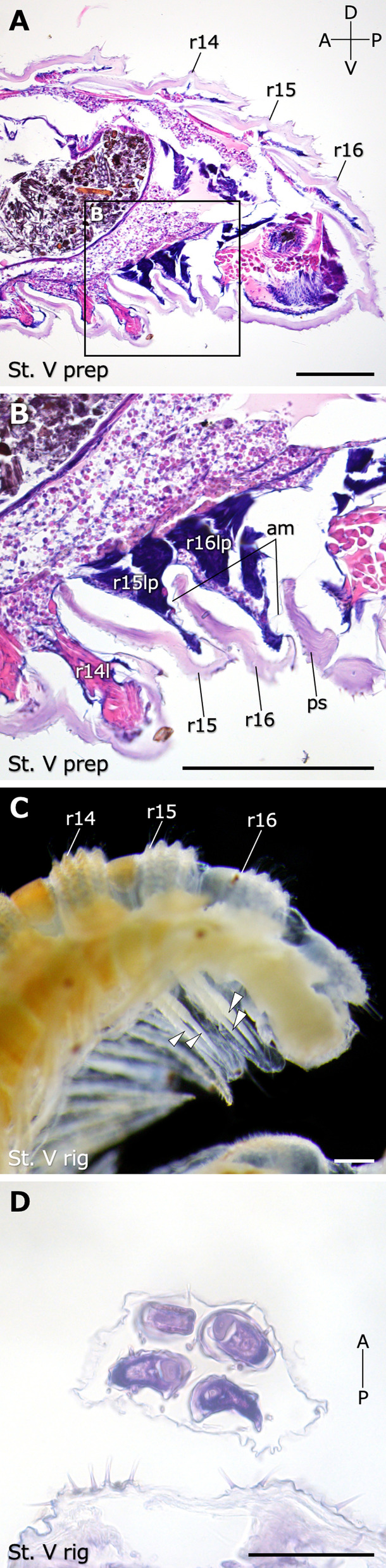
Internal morphologies in stadium V juveniles at the late preparatory and rigidation periods. **A** A histological section of the tail end in the sagittal plane of an individual in the late preparatory period stained with hematoxylin and eosin. **B** Higher magnification of the black boxed region in **A**. **C** The tail end of an individual in the rigidation period fixed in Bouin’s solution. **D** A histological section in transverse planes of a transparent protrusion in the rigidation period. Arrowheads indicate newly added legs elongating in the transparent protrusion. Note that in **C**, only two legs per transparent protrusion are visible because the left and right legs overlap. Scale bars show 200 μm (**A**–**C**), 50 μm (**D**). am, arthrodial membrane; l, legs; lp, leg primordia; ps, preanal sclerite; r, ring (each ring number is added after “r”)

Histological observations on paraffin sections clarified the inner structures of the tail ends (Fig. 3). In stadium V juveniles at the late preparatory period, i.e., 2–3 days before the appearance of the transparent protrusion, epithelial tissues strongly stained by hematoxylin were observed on the ventral side underneath the cuticle of both apodous rings (rings 15, 16) (Fig. 3A, B). These epithelial structures were suggested to be leg primordia. These structures protruded ventrally and posteriorly as expected for legs. A thin membranous structure was observed between the posterior end of ring 15 and the anterior end of ring 16. Similarly, a similar membrane was observed between the posterior end of ring 16 and the anterior end of the preanal sclerite of the telson (Fig. 3B).

In juveniles of stadium V at the rigidation period that were fixed in Bouin’s solution, each transparent protrusion was revealed to contain four legs, i.e., two pairs of legs (Fig. 3C). Cross sections of the transparent protrusion at the rigidation period revealed that two pairs of newly added legs were wrapped in a thin membranous structure (Fig. 3D). The unit composed of two pairs of legs in the transparent protrusion was called a “leg bundle”.

### Morphogenetic process of leg primordia

**Fig. 4:**
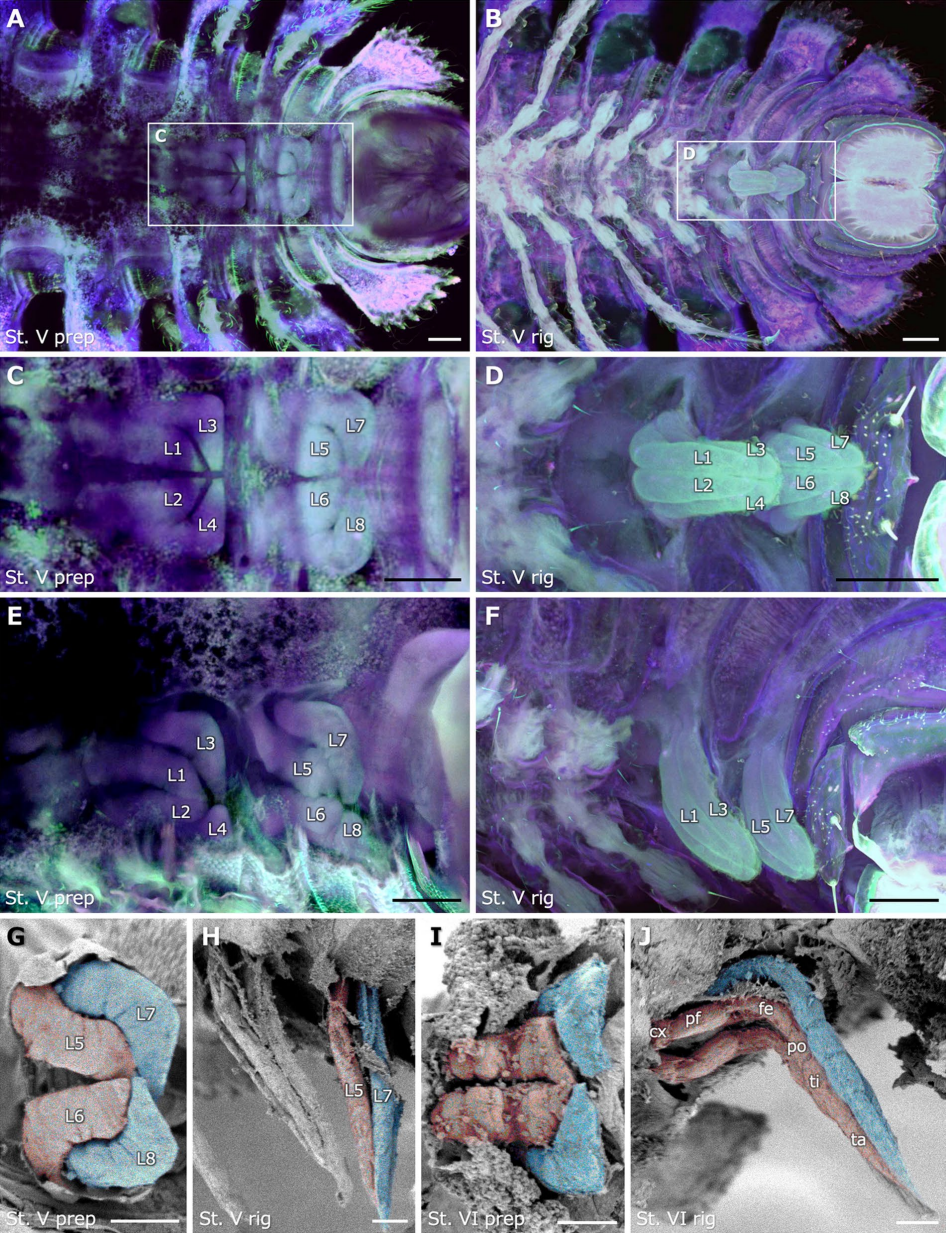
Leg primordia and newly added legs in the late preparatory and rigidation periods. **A**-**F** Autofluorescence images obtained by confocal laser scanning microscope of transparentized samples of stadium V juveniles at 405 (cyan), 488 (green), 561 (red), and 640 nm (blue). **A**,**B** Ventral view of the posterior several rings. **C**,**D** Higher magnification of the white boxed region in **A** and **B**, respectively. **E**,**F** Lateral view of the posterior several rings. **A**,**C**,**E** Leg primordia at the late preparatory period. **B**,**D**,**F** Transparent protrusions at the rigidation period. **G**-**J** SEM images with the covered cuticles removed. **G**,**H** Stadium V juveniles. **I**,**J** Stadium VI juveniles. **G**,**I** Ventral view of two pairs of leg primordia at the late preparatory period. Especially, leg primordia of **G** are of ring 16. **H**,**J** Lateral view of two pairs of newly added legs at the rigidation period. In SEM images, red and blue colorations, respectively, indicate anterior and posterior leg pairs. In **C**-**H**, each leg was labeled L1–8 so that the corresponding leg could be identified. Anterior to the left in all images. Scale bars show 100 μm (**A**-**F**) and 50 μm (**G**–**J**). cx, coxa; fe, femur; pf, prefemur; po, postfemur; ta, tarsus; ti, tibia

To clarify the detailed structures and morphogenesis of leg primordia, the autofluorescence of specimens was observed using confocal laser scanning microscope in four channels simultaneously, 405 nm, 488 nm, 561 nm, and 640 nm. In stadium V juveniles at the late preparatory period, two pairs of leg primordia were observed underneath the cuticle of each apodous ring (Fig. 4A, C, E). Leg primordia were wrinkled and their distal regions were extended to a point on the median line. In stadium V juveniles at the rigidation period, two pairs of newly added legs were observed as a leg bundle (Fig. 4B, D, F), which protruded from a point on the median line.

To observe the surface structure of the leg primordia in more detail, SEM observations were performed, after the covered cuticles were removed, on the stadium V and VI juveniles at the late preparatory and rigidation period. At the late preparatory period, two pairs of wrinkled leg primordia were observed beneath the cuticle in an apodous ring (Fig. 4G, I). The tips of the leg primordia were pointed and the four of them were bundled on the median line. At the rigidation period, two pairs of newly added legs were observed as leg bundle (Fig. 4H, J). Especially in stadium VI juveniles, at least six podomeres were observed in each leg, i.e., coxa, prefemur, femur, postfemur, tibia, and tarsus.

### Newly added rings

**Fig. 5:**
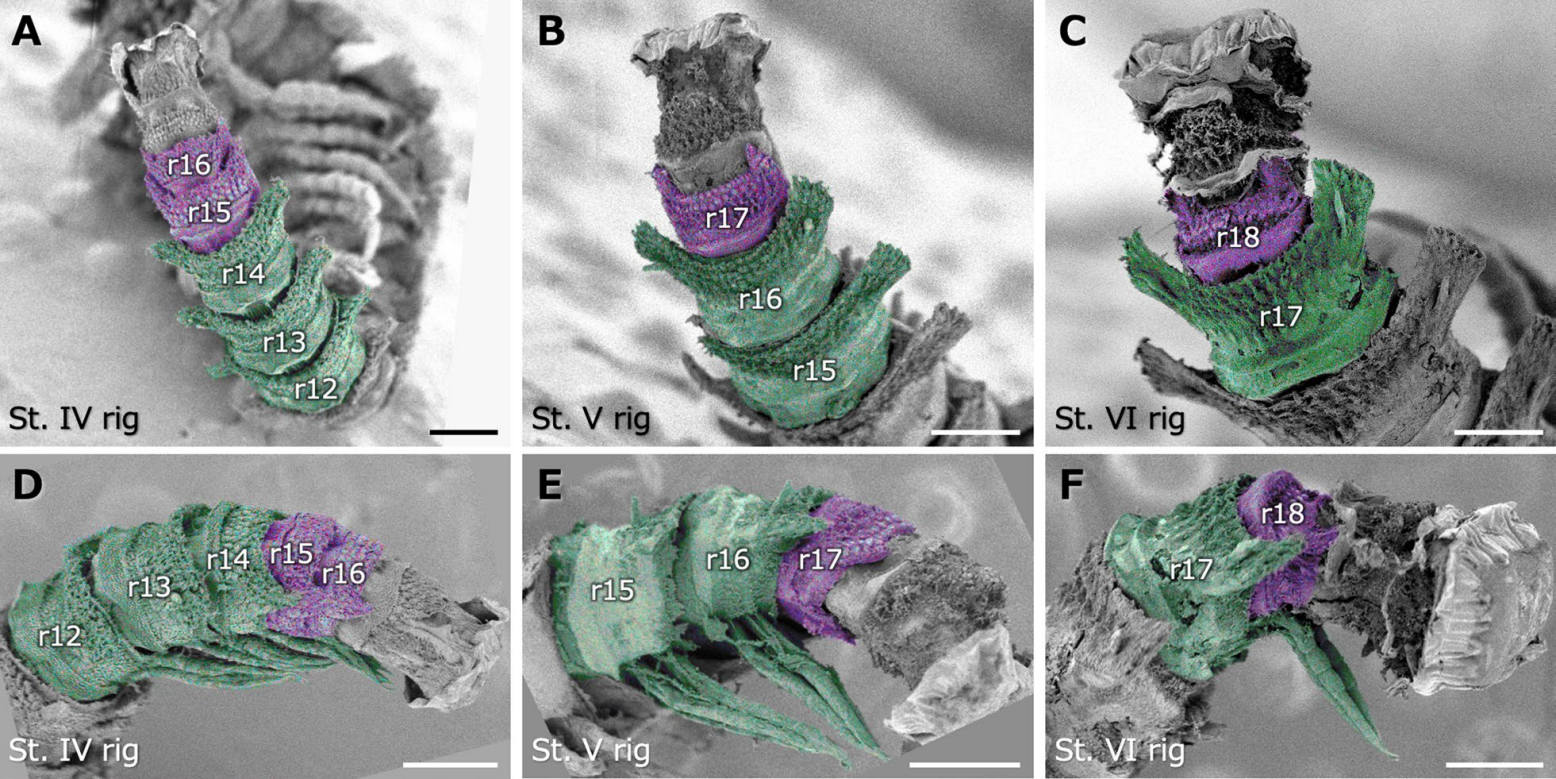
SEM images of newly added rings in the rigidation period with the old cuticles that would be exuviated soon after removed. **A**-**C** Dorsal view, posterior to the upper. **D**-**F** Lateral view, anterior to the left. **A**,**D** Stadium IV juveniles. **B**,**E** Stadium V juveniles. **C**,**F** Stadium VI juveniles. Green and purple colorations, respectively, indicate apodous rings with leg bundles that becomes functional after molting and newly added rings. Scale bars show 200 μm. r, ring (each ring number is added after “r”)

SEM observations on the stadium IV, V, and VI juveniles at the rigidation period with the old cuticle that would be exuviated soon after removed clarified the structure of newly added rings (Fig. 5A–F). In the stadium IV juveniles, the newly added rings, i.e., rings 15, 16, were observed posterior to ring 14. Rings 12–14 were the apparently apodous rings with the leg bundles and would become the newest podous rings after the subsequent molt. The paranota of the newly added rings were still undeveloped and not yet overhanging at the lateral side (Fig. 5A, D). Similar patterns were observed in other stages.

### Observations at the time of molt in *Epanerchodus* sp.

**Fig. 6:**
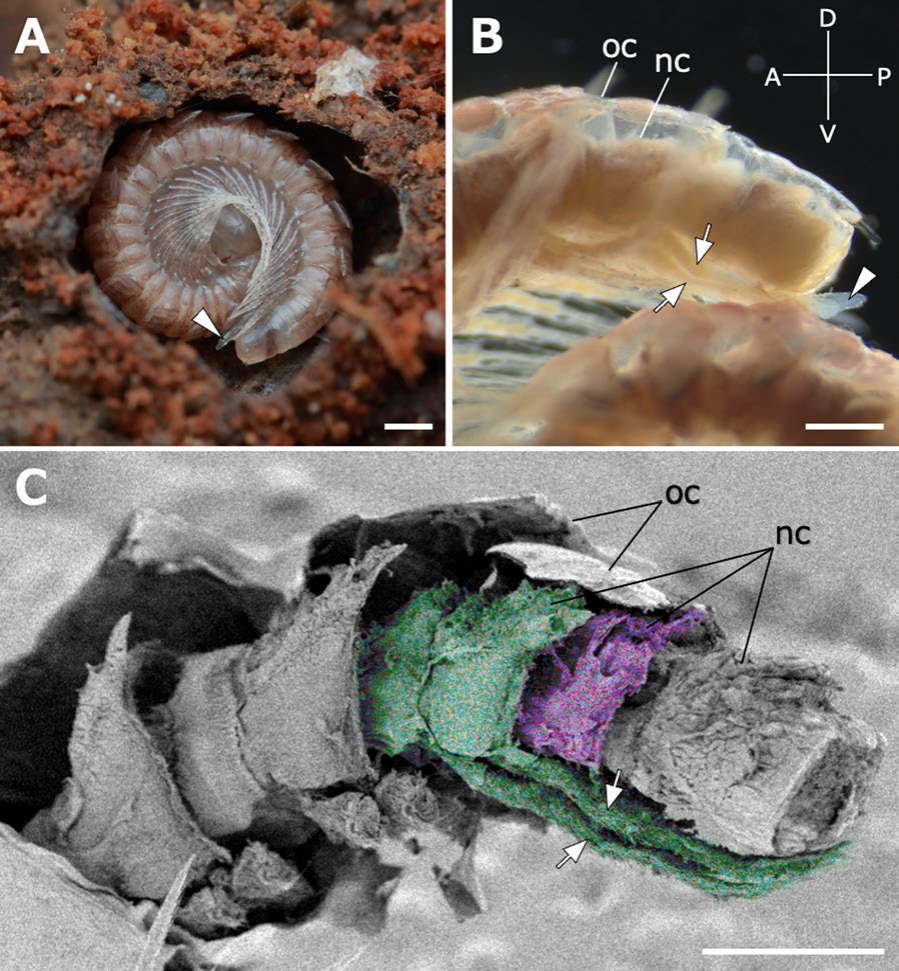
External and internal morphologies of stadium VI juveniles in *Epanerchodus* sp. in the rigidation period. **A** A live individual in a molting chamber of which the covered wall was artificially removed for observation. **B** Lateral view of the posterior several rings of the individual fixed in Bouin’s solution. **C** SEM image of the individual with the cuticles removed. Green and purple coloration, respectively, indicate an apodous ring with forming legs and a newly added ring. Arrows indicate transparent protrusions. Arrowheads indicate newly added legs elongating in the transparent protrusion. Scale bars show 1 mm (**A**), 500 μm (**B**,**C**). nc, new cuticle covering the ring after molting; oc, old cuticle that is later exuviated

In stadium VI juveniles of *Epanerchodus* sp. at the rigidation period, a transparent protrusion was observed on the ventral surface of the apodous ring (Fig. 6A), as in *N. nodulosa*. The stadium VI juveniles of *E*. sp. had one apodous ring and only one transparent protrusion. In the specimen fixed in Bouin’s solution, two pairs of legs, i.e., a leg bundle, were observed in the transparent protrusion (Fig. 6B). SEM observations on the sample in which the covered cuticle was removed clarified that a new ring was added posterior to the apodous ring with a leg bundle (Fig. 6C).

## Discussion

**Fig. 7:**
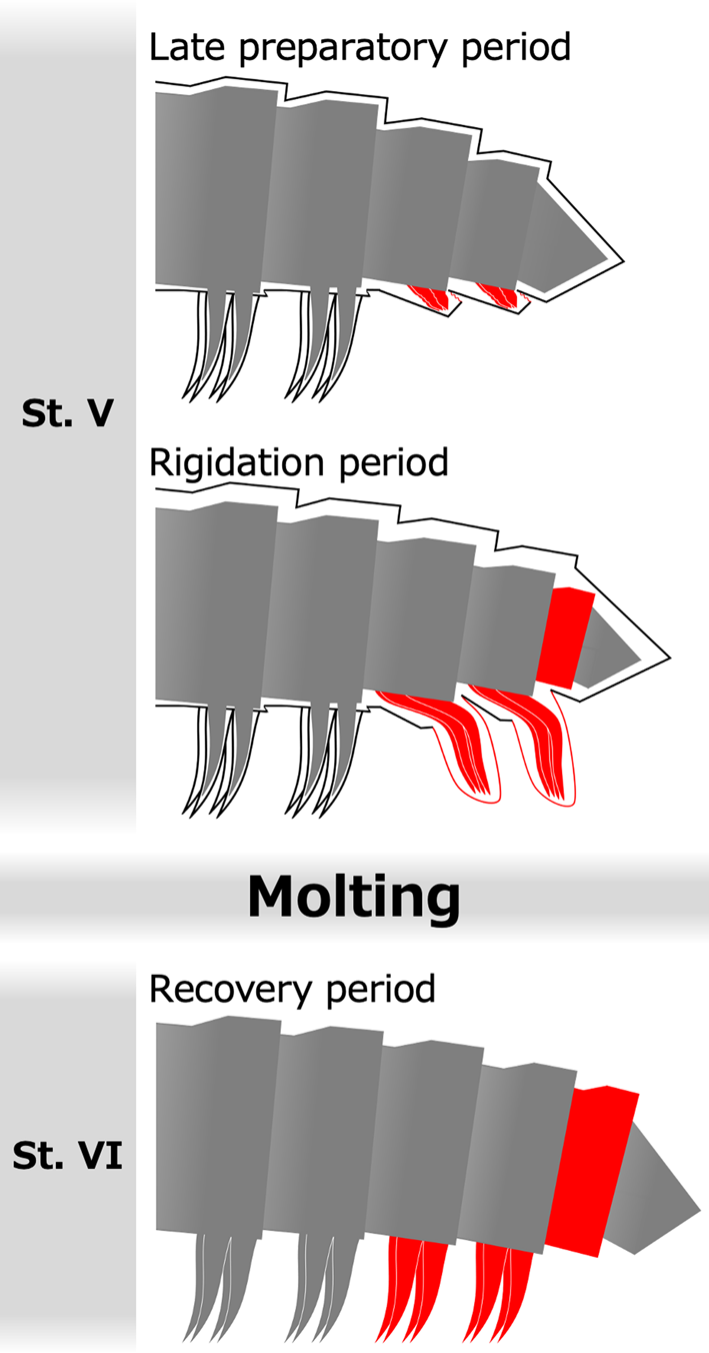
A schematic diagram of the morphogenetic process through a molt in *N. nodulosa*. Newly added legs and rings are indicated in red. This is a representative diagram showing the pattern at stadium V, which is considered to be applicable to other stages

By observing morphogenetic processes at the time of molt in *N. nodulosa*, this study morphologically and histologically revealed the patterns of leg and ring addition associated with molting (Fig. 7). In the juveniles of each stadium, these observations suggested that two pairs of leg primordia are formed inside an apodous ring prior to molt into the next stadium. In the late preparatory period, the leg primordia were wrinkled structures with pointed distal tips (Fig. 4A, C, E, G, I). Histological observations showed that, in contrast to the fully formed legs stained with eosin (see r14l in Fig. 3B), the leg primordia were well stained with hematoxylin, suggesting a dense population of proliferating cells (Fig. 3A, B) [26]. By the rigidation period, these leg primordia have elongated into a transparent protrusion and now project from the apodous ring to lie tightly along the ring posterior to it. A single transparent protrusion contained two pairs of fully segmented legs, i.e., a leg bundle (Figs. 3C, D and 4B, D, F, H, J). In the late preparatory period, deflated membranous structures were observed between an apodous ring and the next one (Fig. 3B). In the rigidation period, transparent protrusions were covered by membranous structures. The membrane appears to derive from an arthrodial membrane that is connected to the apodous ring (Fig. 2M–P). The observations suggested that the wrinkled arthrodial membrane became unfolded as the transparent protrusion elongated from the preparatory period to the rigidation period. In the rigidation period, the newly added ring was formed between the penultimate ring, i.e., the posterior apodous ring, and the telson (Fig. 5A–F). By molting after the rigidation period, the old exoskeleton, including the membrane of the transparent protrusions, is exuviated. Thereby, the podous rings with functional walking legs and the new apodous ring are completely formed. These findings provide morphological and histological evidence for the "law of anamorphosis" proposed by Fabre [20].

In this study, the protrusion was also observed in another species, i.e., *Epanerchodus* sp., just before molting (Fig. 6). A “leg bud” observed in the previous study of *Oxidus gracilis* is also considered to be a transparent protrusion [22]. Taken together, these findings suggest that the process of the protrusion is shared at least among species in Polydesmida. Further studies will be required to verify whether this is a common phenomenon in other millipede lineages.

In general, arthropods can grow and change their morphologies only through molts. However, the discovery of this paper deviates from the general patterns. This study firstly reported, in a millipede, the transparent protrusion appears prior to the anamorphic molt, i.e., before the exuviation, enabling the leg addition on apodous rings. The protrusion accomplished by the expansion of elastic cuticle is suggested to be a unique morphogenetic process that have been specifically modified in the millipede lineage.

Such morphogenetic processes by thin cuticles and not via molting are sometimes seen also in other arthropods. For example, a physogastry seen in Euarthropoda is a phenomenon of extreme inflation of the body trunk for growth during intermolt [27], such as ticks when feeding blood [28], termite queens [29, 30], and larvae of Gnathiidae [31]. In another example, it is known that a head elongation of termites presoldier with soft and flexible cuticles during intermolt [32]. In contrast to these examples, the transparent protrusion observed in the millipedes is quite unique in that a particular part protrudes rapidly and the protrusion is to extend their legs. The morphogenesis by a soft cuticle without molts is suggested to have increased the morphological diversity in arthropods.

Although insects develop by epimorphosis, i.e., the number of segments is constant during postembryonic development, in some holometabolous insects, adult legs are newly formed at the time of metamorphosis. In these cases, imaginal discs, i.e., undifferentiated epithelial cell masses that form adult structures, are formed inside the larval body and protruded during metamorphosis [33, 34]. Such morphogenetic processes in which folded structures expand to form protrusive structures have been widely reported in postembryonic developments of insects [35–37]. In crustaceans, a wrinkled structure is formed during leg regeneration, and this is unfolded through molting [38]. The morphogenetic process of the leg addition in *N. nodulosa* is also similar to the phenomenon seen in these other arthropods and this study is the first report of such morphogenesis in myriapods.

On the other hand, in contrast to the development in holometabolous insects such as *Drosophila*, the development of the leg primordia in *N. nodulosa* shows a unique process, in that leg morphogenesis proceeds largely in a single stage, with the elongation of the leg primordia and its protrusion as a transparent protrusion occurring prior to next molting. This pattern of morphogenesis would be a novel developmental process that has been acquired in some millipede lineages. Although holometabolous insects add their legs through the pupal stage, millipedes do not have such stages. It is possible that in order to efficiently add new legs, millipedes acquired a resting period — they build a molting chamber and rest inside — to accomplish the unique morphogenesis in each stadium.

In *Drosophila,* the elongation of the leg disc is caused by the change in cell shape from anisometric to isometric [34]. It is necessary to clarify whether the leg morphogenesis in *N. nodulosa* is caused by a disc-like system like in *Drosophila*, whether it is accompanied by massive cell proliferation, or whether it is caused by a protrusion of a cell mass that is stored within the body cavity. Such investigations would allow for more detailed comparisons with holometabolous insects.

Anamorphosis has so far been observed in various arthropod lineages and the patterns of segment and leg addition have been described in some species. In Myriapoda, for example, Lithobiomorpha (Chilopoda) develops by hemianamorphosis. In *Lithobius pachypedatus* (Lithobiidae) and *Esastigmatobius longitarsis* (Henicopidae), two molts are required to form the podous segment with functional legs in their anamorphic phase [39, 40]. In crustaceans, isopods develop by hemianamorphosis, with the addition of two segments and one pair of walking legs, i.e., the seventh pereopods. The functional seventh pereopods gradually develop in manca stages through three molts [41–43]. In Chelicerata, both anamorphosis and epimorphosis are known in sea spiders (Pycnogonida) [11]. In *Nymphon brevirostre* which develops by hemianamorphosis, the addition of a segment bearing the walking legs is accomplished over four molts [44]. Combining the findings of these previous studies with the results of this study, it is suggested that, generally in arthropods, multiple molting events would be required to complete podous segments via anamorphosis. This gradual postembryonic process may be a developmental constraint in arthropods. Namely, it may be impossible to add a segment with fully functional legs in a single molt. In a lineage of millipedes (Polydesmida), for example, the first molting produces apodous rings, and the second molting produces legs there. This pattern of gradual addition is probably the most efficient way to produce numerous rings and legs during postembryonic development.

This study revealed the morphogenetic processes only in one millipede lineage, so further detailed observations on the postembryonic development of the millipedes will be required to elucidate the developmental basis underlying the shared mechanisms of anamorphosis. In addition, studies on developmental genetics are very limited in the postembryonic development of myriapods except for a few examples (e.g., [45]). It is possible that genes involved in segmentation and appendage formation during embryogenesis play some roles also in postembryonic development. Since modes of postembryonic development are remarkably diversified in myriapods, elucidation of these processes in myriapods would provide insights into the diversification of body-plan evolution in arthropods.

## Conclusions

This study elucidated the morphogenetic processes of ring and leg addition at the time of molting based on morphological and histological observations during postembryonic development in *N. nodulosa*, showing that two pairs of wrinkled leg primordia in each apodous ring rapidly elongated and protruded as a single transparent protrusion covered by an arthrodial membrane just prior to molt. Furthermore, as previously reported, our results also confirmed that the newly added rings were formed anterior to the telson. The resulting information provides an important basis for a multifaceted understanding of this unique morphogenesis, i.e., the rapid elongation of leg primordia and the appearance of transparent protrusions just prior to molt, that appears to have newly evolved in some millipede lineages, although further studies will be required to verify whether this phenomenon is widespread in other millipedes.

## Methods

### Animals

Stadium IV–VII juveniles of *N. nodulosa* were collected between September 2020 and October 2022 in Iwadono, Higashimatsuyama City, Saitama Prefecture, Japan. The species of collected individuals was identified based on morphological characteristics, described in a previous study [46].

Collected individuals were incubated in plastic containers (about L: 150 × W: 90 × H: 40 mm, 10–20 individuals per container) with a humus mat at 25 °C under constant darkness. Fragments of decaying logs were used as a base for the molting chamber. Water was sprayed at weekly intervals to maintain soil humidity.

For the comparison with other species, morphological observations on *Epanerchodus* sp. (Polydesmida, Polydesmidae) were also carried out. A stadium VI juvenile was collected in May 2022 in Ishizaka, Hatoyama Town, Saitama Prefecture. The identification of species was not possible because it was juvenile and did not have gonopods, a useful identification trait.

### Observations on external morphology

Collected individuals were observed and photographed using a digital camera (Raynox DCR-250 Super MacroScan Conversion lens + Canon EF100mm attached to Canon EOS 8000D) and a stereomicroscope (SZX16; Olympus, Tokyo, Japan) equipped with a digital camera (DP50; Olympus, Tokyo, Japan).

Furthermore, to examine the detailed structures of the ventral surface and leg primordia of the tail ends, observations by scanning electron microscope (SEM) were also carried out. Juveniles in the late preparatory period and the rigidation period were fixed with FAA fixative (ethanol/formalin/acetic acid = 16:6:1) for longer than 24 h and preserved in 70% EtOH for observations by SEM. The samples were dehydrated in increasing concentrations of EtOH and dried using a critical point dryer (Samdri-PVT-3D; Tousimis, Rockville, MD, USA). Dried samples were then coated with gold ions with an E-1010 Ion Sputter (Hitachi, Tokyo, Japan). Ion-coated samples were observed by SEM (JSM-5510LV; JEOL, Tokyo, Japan).

### Histological observations

To histologically observe the inner structures of the tail ends, paraffin sections were made according to the method described in previous studies [47]. Juveniles in the late preparatory period and the rigidation period were fixed in Bouin’s solution (saturated aqueous picric acid solution/formalin/acetic acid = 15:5:1) [48] for longer than 24 h and preserved in 70% EtOH until use. The specimens were dehydrated in increasing concentrations of ethanol, then transferred into xylene, and finally embedded into paraffin. Serial sections (8–10 μm thick) were prepared with a microtome (Spencer Lens Co., Buffalo, NY, USA) and stained with hematoxylin and eosin. Tissues on slides were observed using an optical microscope (BX51; Olympus, Tokyo, Japan) and photographs were taken using a digital camera (DP74; Olympus, Tokyo, Japan) attached to the microscope.

### Autofluorescence observations

To observe detailed inner structures forming under old cuticles, transparentizing and autofluorescence scanning methods were applied to *N. nodulosa* according to the method described in previous studies [49, 50]. Juveniles were fixed in FAA for 2–3 h and preserved in 70% EtOH. To transparentize the samples, they were dehydrated in increasing concentrations of methanol, then sunk into BABB solution (benzyl alcohol/benzyl benzoate = 1:2) overnight at room temperature. Transparentized samples were observed under a confocal laser scanning microscope (FV3000; Olympus, Tokyo, Japan) by irradiating four wavelengths of light: 405 nm, 488 nm, 561 nm, and 640 nm.

### Image processing

Digital images were processed with software GIMP-2.10 (https://www.gimp.org/). Some images photographed with the digital camera (EOS 8000D) were stacked by using imaging software (Zerene Stucker; Zerene Systems, Washington, USA).

## Data Availability

The datasets used and/or analyzed during the current study are available from the corresponding author on reasonable request.
